# Increased apolipoprotein E and decreased TNF‐α in the cerebrospinal fluid of nondemented APOE‐ε4 carriers

**DOI:** 10.1002/npr2.12110

**Published:** 2020-05-19

**Authors:** Daimei Sasayama, Kotaro Hattori, Yuuki Yokota, Ryo Matsumura, Toshiya Teraishi, Sumiko Yoshida, Hiroshi Kunugi

**Affiliations:** ^1^ Department of Mental Disorder Research National Center of Neurology and Psychiatry National Institute of Neuroscience Kodaira Japan; ^2^ Department of Psychiatry Shinshu University School of Medicine Matsumoto Japan; ^3^ Child and Adolescent Developmental Psychiatry Shinshu University School of Medicine Matsumoto Japan; ^4^ Medical Genome Center National Center of Neurology and Psychiatry Kodaira Japan; ^5^ Department of Psychiatry National Center of Neurology and Psychiatry National Center Hospital Kodaira Japan; ^6^ Department of Psychiatry Teikyo University School of Medicine Itabashi‐ku Japan

**Keywords:** alzheimer disease, apolipoproteins E, biomarkers, cerebrospinal fluid proteins, tumor necrosis factor‐alpha

## Abstract

**Aim:**

The ε4 allele of apolipoprotein E gene (APOE) is a well‐known risk factor of late‐onset Alzheimer's disease. However, little is known why this variant confers a risk for Alzheimer's disease. The aim of this study was to examine the influence of the APOE genotype on cerebrospinal fluid (CSF) protein levels.

**Methods:**

The present study performed a secondary analysis on our previously generated database to compare the CSF levels of 1128 proteins between APOE‐ε4 carriers (28 subjects) and noncarriers (104 subjects). All subjects were physically healthy Japanese individuals without dementia.

**Results:**

CSF levels of apoE2, apoE3, and apoE4 were significantly higher (all nominal *P* < 10 × 10^−5^, false discovery rate < 0.001) and those of tumor necrosis factor‐α (TNF‐α) were significantly lower (nominal *P* = 1.39 × 10^−6^, false discovery rate < 0.001) in APOE‐ε4 carriers than in noncarriers. No significant correlation was observed between the CSF levels of TNF‐α and any of the apoE proteins.

**Conclusions:**

Our findings indicate the possible roles of apoE and TNF‐α in the pathogenesis of APOE‐ε4‐associated Alzheimer's disease.

## Abstract 2

The CSF levels of apoE2, apoE3, and apoE4 were higher in APOE‐ε4 carriers. The CSF levels of TNF‐α were significantly lower in APOE‐ε4 carriers. Our findings indicate the possible roles of apoE and TNF‐α in the pathogenesis of APOE‐ε4‐associated Alzheimer's disease.

## Graphical abstract

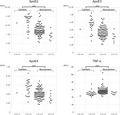

## 1 INTRODUCTION

The apolipoprotein‐E (*APOE*) encodes the protein apolipoprotein E (apoE), which plays a role in lipid transport. Common allelic variants of *APOE* are ε2, ε3, and ε4, which are determined by the two SNPs rs429358 and rs7412. The ε4 allele of *APOE* is the largest genetic risk factor known to date for late‐onset (sporadic) Alzheimer's disease (AD). However, little is known regarding its mechanism of action in conferring the risk for AD.

Cerebrospinal fluid (CSF) biomarkers have been well established for the diagnosis of AD. Low levels of amyloid β_‐42_ and high levels of total tau and phosphorylated tau in CSF have been identified as the core biomarkers for AD.^1^ Several synaptic proteins and inflammatory markers in CSF have also been implicated as possible biomarkers.^2^, ^3^ CSF is practically the only accessible source of proteins derived from the central nervous system (CNS) of living human subjects. Therefore, focusing on CSF proteins might provide an important clue for disentangling the pathophysiological processes of AD. In the present study, we examined the possible association of the *APOE*‐ε4 allele with CSF protein levels in nondemented subjects. This is the second analysis of our CSF proteomics by a genome‐wide genotyping database.^4^

## 2 METHODS

Subjects were 132 Japanese individuals (68 men and 64 women; mean age [standard deviation] = 42.1 [11.7] years) whose data of 1128 CSF proteins and the genotypes of rs7412 and rs4420638 were retrieved from the database of our previous study.^4^ The SNP and protein data from this study can be accessed at NCBI GEO (http://www.ncbi.nlm.nih.gov/geo) under accession number GSE83711. Although the allelic variants of *APOE* are defined by rs429358 and rs7412, the genotype data of rs429358 were not available in our database. Therefore, rs4420638, which is included in the database and is in complete linkage disequilibrium with rs429358 (D′ = 1, *R* ^2^ = .86 in a Japanese population^5^), was used to determine the allelic variants of *APOE.* According to LDlink,^5^ the A allele of rs4420638 is completely associated with the T allele of rs429358, and 89% of the G allele of rs4420638 corresponds to the C allele of rs429358. The numbers of subjects for each predicted genotype of *APOE* are shown in Table 1. *APOE*‐ε4/ε4 or *APOE*‐ε3/ε4 genotypes were considered *APOE*‐ε4 carriers (28 subjects; 13 men and 15 women, mean age = 43.3 [13.9] years), and *APOE*‐ε3/ε3 or *APOE*‐ε2/ε3 genotypes were considered *APOE*‐ε4 noncarriers (104 subjects; 55 men and 49 women, mean age = 41.8 [11.1] years).

**TABLE 1:** Predicted *APOE* genotypes

rs7412	rs4420638	Predicted genotype	Number of subjects
(T;T)	(G;G)	ApoE‐ε1/ε1	0
(T;T)	(G;A)	ApoE‐ε1/ε2	0
(C;T)	(G;A)	ApoE‐ε1/ε3 or ApoE‐ε2/ε4	0
(C;T)	(G;G)	ApoE‐ε1/ε4	0
(T;T)	(A;A)	ApoE‐ε2/ε2	0
(C;T)	(A;A)	ApoE‐ε2/ε3	9
(C;C)	(A;A)	ApoE‐ε3/ε3	95
(C;C)	(G;A)	ApoE‐ε3/ε4	27
(C;C)	(G;G)	ApoE‐ε4/ε4	1

Abbreviation: ApoE: apolipoprotein E.

As described previously,^4^ most subjects had participated in other studies,^6^, ^7^, ^8^ which examined the CSF protein levels in patients with psychiatric disorders (n = 87). All participants were physically healthy and without clinically significant systemic disease. None of the participants were diagnosed with dementia. All subjects were biologically unrelated Japanese individuals. The genotypes were examined using the Illumina HumanOmni1‐Quad BeadChip (Illumina, Inc). The proteomic assessments were performed by SomaLogic Inc as described elsewhere.^9^ The study protocol was approved by the ethics committee at the National Center of Neurology and Psychiatry, Japan. Informed consent for study participation was obtained from every subject.

All statistical analyses were performed using the R software version 3.4.0 (https://cran.r‐project.org/). Differences in age and sex between *APOE*‐ε4 carriers and noncarriers were compared using a *t* test and chi‐square test, respectively. A Mann‐Whitney *U* test was used to compare protein levels between *APOE*‐ε4 carriers and noncarriers. Difference in CSF levels between groups was considered significant at the false discovery rate (FDR) of < 0.01. FDR was calculated according to Benjamini and Hochberg's method. Correlation between CSF protein levels was assessed using Spearman's correlation coefficients.

## 3 RESULTS

**FIGURE 1:**
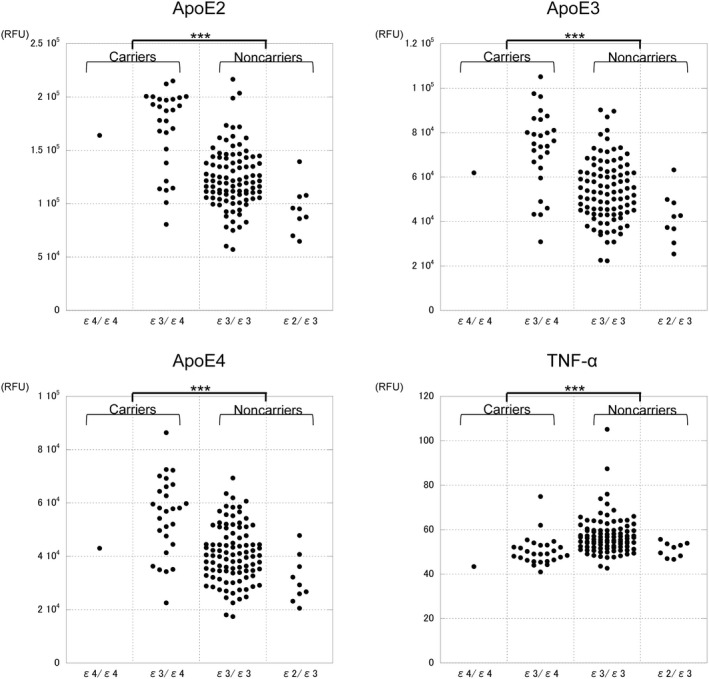
CSF levels in each *APOE* genotype. The CSF levels of apoE2, apoE3, TNF‐α, and apoE4 in each *APOE* genotype are shown. ApoE2, apoE3, and apoE4 were significantly higher in *APOE*‐ε4 carriers than in noncarriers (*P* < .0001, Mann‐Whitney *U* test). TNF‐α was significantly lower in *APOE*‐ε4 carriers than in noncarriers (*P* < .0001, Mann‐Whitney *U* test). The number of subjects in each genotype is as follows. ε4/ε4: healthy subjects (n = 0), psychiatric patients (n = 1). ε3/ε4: healthy subjects (n = 10), psychiatric patients (n = 17). ε3/ε3: healthy subjects (n = 31), psychiatric patients (n = 64). ε2/ε3: healthy subjects (n = 4), psychiatric patients (n = 5). ***P < .0001

The complete results of the Mann‐Whitney *U* test comparing 1128 CSF protein levels between *APOE*‐ε4 carriers and noncarriers are shown in Table S1. The CSF levels of apoE2, apoE3, apoE4, and tumor necrosis factor‐α (TNF‐α) were significantly different between *APOE*‐ε4 carriers and noncarriers after correcting for multiple comparisons using FDR. As shown in Figure 1, apoE2, apoE3, and apoE4 were significantly higher, while TNF‐α was significantly lower in *APOE*‐ε4 carriers than in noncarriers. The CSF levels of the proteins were significantly different depending on *APOE* genotype. The CSF levels of apoE2, apoE3, and apoE4 were significantly correlated with each other (all Spearman's *ρ* > 0.9 and *P* < .0001). However, the CSF levels of TNF‐α were not significantly correlated with any of the apoE proteins (all *P* > .05). As shown in Table 2, the CSF levels of TNF‐α were significantly higher in women than in men (*P* = .040), and apoE2, apoE3, and apoE4 levels showed a significantly negative correlations with age (all *P* < .01).

**TABLE 2:** Associations of CSF protein levels with age and sex

	Total (N = 132)	Men (n = 68)	Women (n = 64)	Mann‐Whitney *U* test comparing men and women	Spearman's correlation with age
Median (LQ, UQ) CSF levels					
ApoE2 [10^3^ RFU]	122 (106, 152)	120 (104, 147)	130 (110, 155)	*U* = 1846, *P *= .13	*ρ* = −0.25, *P *= .003
ApoE3 [10^3^ RFU]	553 (442, 684)	521 (433, 665)	600 (458, 713)	*U* = 1809, *P *= .095	*ρ* = −0.23, *P *= .007
TNF‐α [RFU]	54.2 (50.4, 58.3)	53.5 (49.9, 56.8)	55.0 (51.1, 60.4)	*U* = 1725, *P *= .040	*ρ* = −0.02, *P *= .82
ApoE4 [10^3^ RFU]	414 (345, 518)	398 (330, 504)	440 (353, 543)	*U* = 1760, *P *= .058	*ρ* = −0.23, *P *= .008

Abbreviations: ApoE, apolipoprotein E; CSF, cerebrospinal fluid; LQ, lower quartile; RFU, relative fluorescent units; TNF, tumor necrosis factor; UQ, upper quartile.

Because the study subjects included 87 patients with psychiatric disorders, we next compared the CSF protein levels between *APOE*‐ε4 carriers and noncarriers separately in psychiatric patients and psychiatrically healthy subjects to examine whether a psychiatric disorder influenced the effect of *APOE* genotypes on CSF protein levels. Mann‐Whitney *U* tests showed that CSF levels of apoE2, apoE3, and apoE4 were significantly higher in *APOE*‐ε4 carriers than in noncarriers in psychiatric patients (all *P* < .001) as well as healthy subjects (all *P* < .01). Furthermore, TNF‐α levels were significantly lower in *APOE*‐ε4 carriers than in noncarriers in psychiatric patients (*P* < .001) and healthy subjects (*P* = .031). Thirty‐one patients were prescribed antipsychotics. The daily chlorpromazine‐equivalent dose of antipsychotics did not significantly correlate with the CSF levels of apoE2, apoE3, apoE4, or TNF‐α (all *P* > .05).

## 4 DISCUSSION

The present study is the first proteome‐wide analysis to examine the influence of *APOE*‐ε4 alleles on CSF protein levels in nondemented subjects. Our findings showed that carrying the *APOE*‐ε4 allele is associated with increased apoE2, apoE3, and apoE4, as well as decreased TNF‐α levels in the CSF. The difference between *APOE*‐ε4 carriers and noncarriers remained robustly significant even after correcting for multiple comparisons.

Contrary to our findings, some previous studies reported no significant difference in total CSF apoE concentrations between *APOE*‐ε4 carriers and noncarriers.^10^, ^11^, ^12^ Furthermore, Cruchaga et al^13^ reported that the total apoE levels in the CSF were lower in *APOE*‐ε4 carriers than in noncarriers, irrespective of the presence of AD. However, consistent with our findings, Darreh‐Shori et al^14^ reported that CSF apoE protein levels were higher in patients with AD carrying the *APOE*‐ε4 allele, in an ε4 dose‐dependent manner. One reason for the inconsistencies between study results may be the small number of subjects in some studies reporting negative findings.^11^, ^12^ Another reason may be the difference in the apoE quantification methods. Indeed, Cruchaga et al^13^ reported a low correlation between apoE levels measured in different assays. However, we believe that our findings are reliable because the CSF protein levels recorded in the database were measured by a highly sensitive analysis using SOMAmer reagents with high affinity and specificity for their target proteins.^15^ The low intra‐assay coefficient of variation previously reported for SOMAscan assay (6.1%, 5.8%, 8.1%, and 2.5% for apoE2, apoE3, apoE4, and TNF‐α, respectively^15^) also indicates the good precision of the assay system. Furthermore, this study is useful in that it is the first, to our knowledge, to examine the CSF levels of each apoE isoform to investigate the association with the *APOE* genotypes.

The significantly negative correlations observed in the present study between age and each apoE level contradict with previous findings^13^, ^14^ showing a positive correlation between age and CSF apoE levels. A possible explanation for the inconsistency may be that we did not include patients with AD in our study. Future studies are necessary to elucidate the influence of aging on the CSF levels of apoE.

The present study is also the first, to our knowledge, to report that TNF‐α levels in the CSF are decreased in *APOE*‐ε4 carriers. Our result seems to contradict previous findings that TNF‐α plays a role in the pathophysiology of AD by enhancing amyloid beta production.^16^ Indeed, a previous study reported that TNF‐α was elevated in the blood and CSF of patients with AD.^17^ However, in vitro data suggest that TNF‐α may exert a neuroprotective effect by stimulating neuronal cells to express bcl‐2,^18^ which may explain the elevated risk of developing AD in *APOE*‐ε4 carriers with decreased TNF‐α levels. Further investigations are necessary to clarify the role of decreased TNF‐α in increasing the risk for AD in *APOE*‐ε4 carriers.

Regarding the association with apoE, TNF‐α secretion induced by lipopolysaccharide stimulation was inhibited by preincubation with apoE in glial cells.^19^ Conversely, ApoE gene‐knockout in mice resulted in high expressions of cytokines, including TNF‐α in the brain.^20^ These findings are consistent with our results that subjects with high apoE‐expressing genotypes had lower CSF TNF‐α levels. However, no significant correlation between apoE and TNF‐α levels was observed. Several possibilities may be considered for the lack of significant correlation between apoE and TNF‐α levels; for example, *APOE*‐ε4 alleles may function to decrease TNF‐α levels through a mechanism independent of apoE proteins, or TNF‐α levels may not adequately reflect the fluctuating levels of apoE proteins, thereby failing to achieve statistical significance.

Several limitations must be considered when interpreting our findings. First, subjects included patients with psychiatric disorders. However, analyzing psychiatric patients and psychiatrically healthy subjects separately also resulted in same findings. Therefore, the influence of psychiatric disorders in a portion of the subjects may be minimal for apoE2/3/4 and TNF‐α proteins, although a possibility remains that psychiatric disorder or medication in these subjects contributed to negative results in other proteins. Secondly, the number of homozygotes for *APOE*‐ε4 was so small (n = 1) that we could not examine whether the association occurs in a dose‐dependent manner.

In conclusion, *APOE*‐ε4 alleles were associated with increased apoE2, apoE3, and apoE4, as well as decreased TNF‐α levels in the CSF of nondemented individuals. Further investigations are warranted to elucidate the role of TNF‐α and apoE proteins in the development of *APOE*‐ε4‐associated AD.

## CONFLICT OF INTEREST

None to declare.

## AUTHOR CONTRIBUTIONS

DS and HK designed the study, and DS wrote the draft of the manuscript. KH, YY, RM, TT, and SY retrieved data from the database used in the present study. DS and HK performed statistical analyses. HK supervised the data analysis and writing of the paper. All authors contributed to and have approved the final manuscript.

## APPROVAL OF THE RESEARCH PROTOCOL BY AN INSTITUTIONAL REVIEWER BOARD

The study was approved by the Ethics Review Committee of National Center of Neurology and Psychiatry, Japan.

## INFORMED CONSENT

Informed consent for study participation was obtained from every subject.

## Supporting information

Table S1

## Data Availability

The data that support the findings of this study are openly available in NCBI Gene Expression Omnibus at http://www.ncbi.nlm.nih.gov/geo, reference number GSE83711.
